# Reactive Dicarbonyl Scavenging with 2-Hydroxybenzylamine Improves MASH

**DOI:** 10.3390/nu17040610

**Published:** 2025-02-07

**Authors:** Joyce Cheung-Flynn, John A. Rathmacher, Lisa M. Pitchford, Yanhua Xiong, Charles Robert Flynn

**Affiliations:** 1 Department of Vascular Surgery, Vanderbilt University Medical Center, Nashville, TN 37232, USA; joyce.cheung-flynn@vumc.org; 2 MTI Biotech Inc., Iowa State University Research Park, Ames, IA 50010, USA; rathmacher@mtibiotech.com (J.A.R.); pitchford@mtibiotech.com (L.M.P.); 3 Department of Animal Science, Iowa State University, Ames, IA 50011, USA; 4 Department of Surgery, Vanderbilt University Medical Center, Nashville, TN 37232, USA

**Keywords:** fatty liver disease, fibrosis, steatohepatitis

## Abstract

**Background:** Products of lipid peroxidation include a number of reactive lipid aldehydes including reactive dicarbonyl electrophiles (DEs) and contribute to disease processes. DEs play a significant role in the development and progression of metabolic-associated steatotic liver disease (MASLD) by contributing to oxidative stress, inflammation, protein dysfunction, and mitochondrial impairment. Reducing DE stress may be a potential strategy for managing MASLD. We hypothesized that the DE scavenger 2-hydroxybenzylamine (2-HOBA) would reduce liver injury by reducing liver protein adduct formation by DE in mouse models of MASLD. **Methods:** Protein adducts were measured in human livers by immunohistochemistry and immunoblot. The effects of 2-HOBA were assessed in two different mouse models of MASLD. **Results:** Isolevuglandin (IsoLG) protein adducts were increased in MASH-staged human livers relative to histologically normal controls. Diet-Induced Animal Model of Nonalcoholic Fatty Liver Disease (DIAMOND) mice treated with 2-HOBA exhibited significantly lower fibrosis scores (* *p* = 0.012) and reduced liver transaminases (AST, *p* = 0.03) and ALT, *p* = 0.012) by over 40%. In STAM (Stelic Animal Model) mice, 2-HOBA improved NAFLD activity scores (*p* = 0.03, NAS), hyperglycemia, and inflammatory cytokines and reduced serum F2-isoprostanes (IsoPs) by 30%, *p* = 0.05. These improvements were absent mRNA changes in hepatic antioxidant enzymes (*Cat*, *Gpx1*, or *Sod2*) or ROS-generating proteins (p22PHOX, p47PHOX, NOX4 or COX1). **Conclusions**: DE scavenging with 2-HOBA may be a promising therapeutic strategy for managing MASLD. While findings are currently limited to male mice, a nutraceutical that reduces liver fibrosis could significantly improve the management of MASH by offering a non-invasive treatment option to potentially slow or reverse liver scarring, delay progression to cirrhosis, and improve patient outcomes, while also providing a potential treatment option for patients who may not be suitable for other interventions like liver transplantation.

## 1. Introduction

Metabolic-associated steatotic liver disease (MASLD) is intricately linked to metabolic syndrome, with obesity, high serum triglycerides, and hyperglycemia as key contributors. Notably, MASLD also occurs in lean individuals, particularly in Asian populations, with prevalence estimates ranging from 8 to 23% [1]. The progression to Metabolic-Associated Steatohepatitis (MASH) involves complex pathophysiological processes, including insulin resistance and hyperinsulinemia, which exacerbate hepatic steatosis [2,3], and increased production of reactive oxygen species (ROS) in the liver through mitochondrial β oxidation and dysfunction, inflammation, and metal redox cycling [4]. Despite the concerning epidemiologic trends, targeted medical treatments for MASH remain limited. Resmetirom (Rezdiffra^®^) only recently emerged as the sole targeted medical treatment for MASH [5]. Resmetirom reduces liver fat by enhancing hepatic β-oxidation upon partial activation of thyroid hormone receptor β [6]. Unfortunately, over half of MASH patients taking Resmetirom did not show any improvement in their condition, underscoring a need for additional and adjunctive therapies.

MASLD progresses to MASH though a complex series of mechanisms that involve lipid accumulation, oxidative stress, and inflammation. Increased insulin resistance and hyperinsulinemia exacerbate hepatic steatosis by diverting sugars from glycogen synthesis to de novo lipogenesis and by inhibiting lipolysis [3]. Increased ROS production in the liver occurs through mitochondrial β oxidation and dysfunction, inflammation, and metal redox cycling [4]. Lipid peroxidation is considered a key contributor to liver damage in MASLD, where excessive oxidative stress leads to the breakdown of lipids within liver cells, generating harmful compounds that promote inflammation and fibrosis [7,8]. Insulin and lipid peroxidation have an inverse relationship, meaning that high levels of insulin can lead to decreased lipid peroxidation, while conditions causing insulin resistance often result in increased lipid peroxidation, contributing to further insulin resistance through damage to cellular components involved in insulin signaling pathways; essentially, when insulin signaling is impaired, the body can experience increased oxidative stress and lipid peroxidation, creating a vicious cycle. Furthermore, lipid peroxidation by-products can directly impair insulin receptor signaling by modifying key proteins within the pathway, further hindering glucose uptake and exacerbating insulin resistance. ROS-catalyzed lipid peroxidation generates highly reactive dicarbonyl electrophiles (DEs), including 4-oxo-2-nonenal, malondialdehyde (MDA), and isolevuglandins (IsoLGs). The most reactive is IsoLGs, which are highly reactive toxic products of lipid peroxidation and prostaglandin endoperoxide rearrangement. They rapidly react with primary amines on lysine residues in proteins [9,10]. IsoLGs also react with phosphatidylethanolamines (PEs) in lipoproteins and lipid membranes [11,12] and dC bases in DNA [13]. IsoLG-protein adducts disrupt various cellular processes, including protein folding, oligomerization, binding, proteasome degradation, and promotion of inter-molecular crosslinking and stimulate immune responses [14]. Previous research has shown elevated IsoLG-modified high-density lipoprotein (HDL) in MASH patients [15]. Additionally, IsoLGs activate hepatic stellate cells in vitro, leading to increased MAPK activation (ERK/JNK), cytokine and chemokine production, associated with intracellular ROS production, and autophagy induction. These findings link oxidative injury from IsoLGs to disease progression in fibrotic liver diseases [16].

There are no large-scale clinical studies definitively proving that directly targeting lipid peroxidation is effective in reducing the disease burden of MASLD or MASH. While some clinical trials have explored the use of antioxidants in MASLD, the results have been mixed, with limitations like small sample sizes and lack of specific biomarkers to directly measure lipid peroxidation in the liver. One compound, pentoxifylline, has been demonstrated to decrease the oxidized lipid products 9-hydroxy-octadecadienoic acid (9-HODE) and 13-oxo-octadecadienoic acid (13-oxoODE) in NASH [17]. However, other studies examining the liver-protecting effects of other antioxidants, such as vitamin E, in MASLD did not see correlations between 9-HODE and 13-HODE with hepatic 4-HNE protein adduct levels after treatment, suggesting that these oxidized lipids may reflect systemic and not liver-specific lipid peroxidation [18]. Of note, lipid peroxidation in NAFLD (now referred to as MASLD) has been studied primarily by examining peroxidation markers in circulation and not in liver biopsies [19,20].

**Figure 1:**
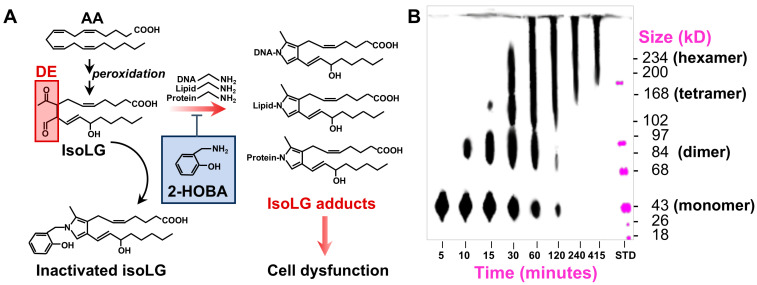
Multiple oxidized lipid peroxidation protein products (OxLPPs), including isolevuglandins (IsoLGs), once formed, adduct with liver lipids, DNA, and proteins. (**A**) Dicarbonyl electrophiles (DEs) react with lysine or other primary amines to form Schiff base and pyrrole adducts. 2-HOBA prevents adduct formation. (**B**) Gel electrophoresis of DE-adducted chicken albumin over time showing progression of protein cross-linking and aggregate formation.

2-Hydroxybenzylamine (2-HOBA) is a natural product of buckwheat seeds that acts as a selective DE scavenger reacting ~1600-fold faster with IsoLGs than lysine, preventing molecular adduction in vitro and in vivo (Figure 1), as previously described [21]. 2-HOBA has been shown to be non-toxic, with proven safety in humans and animals [22,23,24] and with demonstrated efficacy in reducing atherosclerosis, lipid peroxide formation, and inflammation in the *Ldlr*^−/−^ mouse model [25]. 2-HOBA has also shown promise in preclinical models for other indications including hypertension [26], atrial fibrillation [27], systemic lupus erythematosus [28], pulmonary hypertension [29], gastric cancer [30], Alzheimer’s disease [31], and hypertension-induced end-organ damage [32].

This study aims to assess the relative abundance of IsoLG adducts in human livers staged for MASLD and to evaluate the efficacy of 2-HOBA in mitigating MASH progression in two pre-clinical mouse models, a diet-induced animal model of MASLD (DIAMOND), and the Stelic Animal Model (STAM). These models were chosen for their ability to exhibit histopathological features of human MASLD and MASH and display progressive disease courses that recapitulate MASH development from simple steatosis (MASL) to advanced steatohepatitis, cirrhosis, and hepatocellular carcinoma (HCC). By investigating the role of IsoLG adducts and the potential of 2-HOBA as a therapeutic agent, this study aims to contribute to the development of adjuvant novel strategies for MASH treatment and prevention.

## 2. Methods

### 2.1. Human Studies

Wedge liver biopsies of the left lateral lobe of the liver were obtained during elective bariatric surgery. Liver histology was reported by a minimum of two pathologists with experience in reporting hepatic histopathology. Hepatic steatosis, ballooning, lobular inflammation, and fibrosis were reported in accordance with the NASH Clinical Research Network (NASH-CRN) scoring criteria [33]. Definite histological MASH was defined as NAFLD activity score (NAS) ≥ 5. Inclusion criteria included scheduling for bariatric surgery, obesity ≥ 40 kg/m^2^ or ≥35 kg/m^2^, and one comorbidity (type 2 diabetes [fasting blood glucose ≥ 120 mg/dL; HbA1C ≥ 6.5%], known fatty liver disease, hypertension, cardiovascular disease, or hyperlipidemia). Patients were excluded if they had prior bariatric surgery, malignancy (<5 years), known history of intestinal disease, malabsorptive syndrome, established organ dysfunction, renal disease, alpha 1 ant-trypsin disease, Wilson’s disease, viral hepatis, alcoholic liver disease, were pregnant or breastfeeding, or smoked > 7 cigarettes per day. The study adhered to the following ethical guidelines as approved by the Internal Review Board of Vanderbilt University (090657 and 171845) and registered at ClinicalTrials.gov (NCT00983463 and NCT03407833). Studies were conducted in accordance with NIH and institutional guidelines for human subject research and conformed to the ethical guidelines of the 1975 Declaration of Helsinki; subjects provided informed written consent.

### 2.2. Animal Studies

All experiments followed established guidelines for the care and use of laboratory animals [34] and were approved by the VUMC Institutional Animal Care and Use Committee (IACUC) (M/13/257). Animals were housed under 12 h light/darkness cycles at 23 ± 2 °C and 45 ± 10% relative humidity, with ad libitum access to food and water. There were no interventions taken to address differences in diet intake, water consumption, or physical activity between treatment and control groups. Sample sizes of 14 mice per strain with a 1:1 control to 2-HOBA allocation ratio were calculated to have a power of 0.96 to detect a 48% reduction (effect size d of 2.20) in ALT based on a similar study examining the effects of pentoxifylline on NAFLD in obese mice [35]. However, practical considerations in animal costs and availability limited sample sizes to 12 mice per study (n/6 group), yielding a power of 0.94 (assuming the same effect size), which we deemed acceptable.

The DIAMOND model mice included 12 male mice (3-week-old), obtained from Sanyal Biotechnology (Chesterfield, VA, USA). The mice were quarantined for 7 weeks and screened for pathogens and parasites as part of standard institutional quarantine procedures. During this time, their diet included normal chow + Fenbendazole (150 mg/kg; ENVIGO TD.01432) from 3 weeks to 8 weeks of age. The mice were then switched to a high-fat diet (ENVIGO TD.02183, 42% fat chow + Fenbendazole 150 mg/kg) plus sugar water (23.1 g/L fructose and 18.9 g/L glucose in 10 L water) from 8 to 24 weeks of age. Treatment groups (from 12 weeks) consisted of control (*n* = 6) or 2-HOBA (*n* = 6) included in drinking water (1 g/L). The mice were sacrificed at the end of 24 weeks, and tissues were harvested.

STAM: Fourteen-day pregnant C57BL/6 dams were purchased from Japan SLC, Inc. (Hamamatsu, Japan). Male mice from each litter (*n* = 12 total) were administered a single subcutaneous injection of streptozotocin (200 μg; STZ; Sigma-Aldrich, St. Louis, MO, USA) 48 h after birth to induce MASH [36]. At 3 weeks of age, mice were divided into two groups: vehicle control (*n* = 6), and 2-HOBA (*n* = 6, 1 g/L in drinking water). The diet consisted of ad libitum high-fat diet (HFD32, CLEA Japan, Tokyo, Japan) from 3 weeks of age for 6 weeks (sacrifice at 9 weeks of age). Mice in the 2-HOBA group received 2-HOBA in drinking water (1 g/L), while the vehicle control group received plain water without 2-HOBA. Body weight and food/water intake were monitored weekly. Animals were sacrificed at 9 weeks of age following 6 weeks of 2-HOBA or vehicle treatment.

### 2.3. Serum and Plasma Analyses

Serum levels of alanine transaminase (ALT), aspartate transaminase (AST), and gamma-glutamyl transaminase (GGT) were measured by the Vanderbilt Tissue Pathology Core (ACE Alera chemistry, Alfa Wasserman, West Caldwell, NJ, USA). Cytokines (IL-1α, IL-6, IL-1β, IL-10, IL-17, MCP-1, TNFα) were measured using the MILLIPLEX_MAP_ Mouse Metabolic Hormone Magnetic Bead Panel immunoassay (MMHMAG-44K; Millipore, Billerica, MA, USA). Oxidative stress markers of IsoPs [37] and IsoLGs [38] were measured as previously described using mass spectrometry employing [^2^H_4_]8-iso-PGF2α [37] and purified ^13^C γ-KA, [9], respectively, as internal standards. Anti-γ-KA titers, an index of adaptive immune response, were measured using a single-chain monoclonal antibody against IsoLG-lysine adducts independent of protein backbone [39]. Plasma 2-HOBA was measured as previously described [24].

### 2.4. Murine Histopathological Analyses

Formalin-fixed, paraffin-embedded liver sections, stained with hematoxylin/eosin (H/E) or picrosirius red (for fibrosis), were scored blindly by an experienced pathologist, with scoring criteria of steatosis (0–4), ballooning (0–3), inflammation (0–4), and fibrosis (0–4).

### 2.5. Immunoblotting

We performed immunoblotting on tissue preparations using snap-frozen liver samples homogenized in lysis buffer (150 mM NaCl, 50 mM tris pH 8, 1% NP40, 0.5% deoxycholate, 5 mM EDTA, 5 mM EGTA), protease inhibitor cocktail I (1:100 dilution, Sigma Aldrich, St. Louis, MO, USA), and phosphatase inhibitor cocktails II and III (1:100 dilutions each, Sigma Aldrich) and then pelleted. Protein separation was carried out using 4–12% gradient polyacrylamide gels with MOPS buffer (Invitrogen). Primary antibodies raised in rabbit against AKT (#9272), p-AKT Ser473 (#9271), GSK3β (#5676), and p-GSK3β Ser9 (#5558) (Cell Signaling Technologies, Danvers, MA, USA) were used at 1:1000 dilution. Antibodies raised against IsoLG-lysyl adduct (D11) were diluted in 1% polyvinylpyrrolidone blocking buffer and incubated at room temperature for 1 h as previously described [39]. Secondary antibodies, including fluorescently labeled goat anti-rabbit and donkey anti-goat (Li-Cor Inc., Lincoln, NE, USA), were diluted 1:10,000 and incubated in blocking buffer at room temperature for 1 h with shaking. Imaging was performed using the Odyssey Infrared Imaging System (Li-Cor Inc.) as previously described [39].

### 2.6. RNA Isolation

Liver tissues were lysed in RLT lysis buffer and RNA were isolated using the RNeasy Mini Kit (Qiagen, Germantown, MD, USA; #74101) following the manufacturer’s protocol. RNA quality was determined using the Agilent Bioanalyzer (Agilent Technologies, Santa Clara, CA, USA). Total RNA yield and 260/280 ratios were measured using a NanoDrop spectrophotometer (Thermo Scientific, Waltham, MA, USA).

### 2.7. Quantitative Real-Time Polymerase Chain Reaction

Total RNA was reverse-transcribed into complementary DNA with the SuperScript VILO cDNA synthesis kit (Invitrogen, Carlsbad, CA, USA, #11754050). Quantification of the complementary DNA template was performed by real-time PCR using SYBR green fluorescence on a Bio-Rad CFX96 thermal cycler (Bio-Rad Laboratories, Hercules, CA, USA). Expression was normalized to that of 18S as an internal control. We used the 2^−ΔΔCt^ method to determine the relative RNA levels. The primers used in this study were 18S-F 5′-GTAACCCGTTGAACCCCATT-3′, 18s-R 5′-CCATCCAATCGGTAGTAGCG-3′, Cat-F 5′-ATTGCCGTCCGATTCTCC-3′, Cat-R 5′-CCAGTTACCATCTTCAGTGTAG-3′, Gpx1-F 5′-GCTGCTCATTGAGAATGTCG-3′, Gpx1-R 5′-GAATCTCTTCATTCTTGCCATT-3′, Sod2-F 5′-GCTGCACCACAGCAAGCA-3′, Sod2-R 5′-TCGGTGACGTTCAGGTTGTTC-3′, Nox4-F 5′-TCATGGATCTTTGCCTGGAGGGTT-3′, Nox4-R 5′-AGGTCTGTGGGAAATGAGCTTGG A-3′, p22phox-F 5′-TGTTGCAGGAGTGCTCATCTGTCT-3′, p22phox-R 5′-AGGACAGCCC GGACGTAGTAATTT-3′, p47phox-F 5′-AGGTTGGGTCTGCATCCTATTT-3′, p47phox-R 5′-TGGTTACATACGGTTCACCTGCGT-3′, Cox1-F 5′-AGGGCTGGCCGGATTG-3′, and Cox1-R 5′-CAGCCACATGCAGAACATGAT-3′.

### 2.8. Statistics

All data are expressed as mean ± standard error. Unless otherwise indicated, one-way analysis of variance with Dunn’s post-test was used to compare three or more groups, while either an unpaired Student’s *t*-test (two-tailed) or Mann–Whitney U-test (two-tailed) was used for binary comparisons. The t-test was applied to data with a normal distribution, while the Mann–Whitney U-test was used for non-normally distributed data. A *p*-value of ≤0.05 was considered statistically significant. Robust Regression Outlier Testing (ROUT) with false discovery rate Q set at 1% was used to detect outliers. Prism 10.1.2 (GraphPad, San Diego, CA, USA) was used for data analysis.

## 3. Results

### 3.1. Lipid Adducts in MASLD

**Figure 2:**
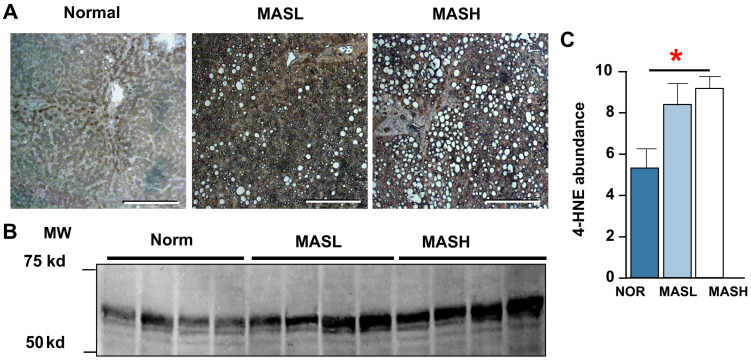
Liver protein adducts increase with MASLD severity. (**A**) Immunohistochemistry detecting IsoLG-lysyl adducts in normal, MASL, or MASH human liver. Scale bar = 200 μm. (**B**) Immunoblot and (**C**) quantitation of 4-HNE-adducted proteins (absorbance units per microgram protein) in MASLD human livers. * *p* ≤ 0.05 by 1-way ANOVA. Data are expressed as mean ± SEM.

We recently reported that high-density lipoprotein (HDL) isolated from MASH patients showed higher levels of IsoLG adducts and elicited greater inflammatory responses in vitro (IL-1β and TNFα) compared to HDL from non-MASH patients [15]. In the current study, we used immunohistochemistry (IHC) to probe normal, MASL, and MASH liver biopsies with a D11 antibody specific to IsoLG-lysyl adduct epitopes. Our results show robust cross-reactivity in MASL and MASH samples compared to normal liver tissue (Figure 2A, B), with the highest antibody reactivity in hepatocytes and fenestrated endothelial cells lining liver sinusoids. We also quantified 4-oxo-2-nonenal (4-HNE) protein adducts in human liver specimens by immunoblot. 4-HNE adduct levels increased progressively with worsening MASLD severity ranging from 5.08 ± 0.73 in histologically normal specimens to 8.01 ± 0.85 in MASL and 8.8 ± 0.46 AU/μg protein in MASH (Figure 2C, *p* = 0.021).

### 3.2. Effects of 2-HOBA in DIAMOND Mice

**Figure 3:**
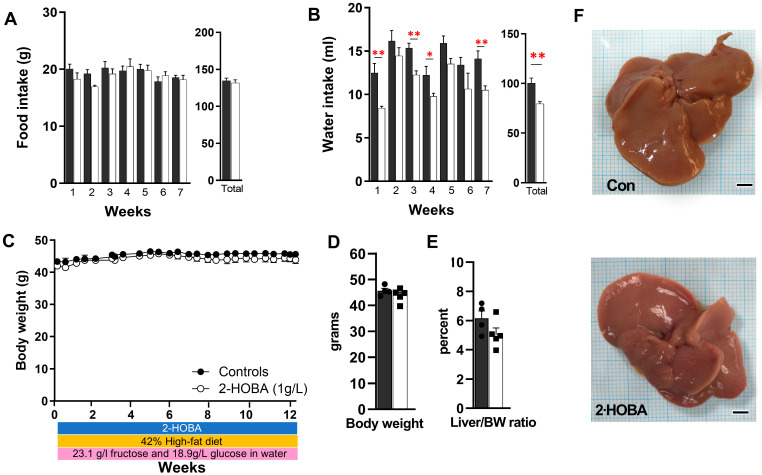
Food intake and water intake in DIAMOND mice treated with 2-HOBA. (**A**) Food intake and water intake (**B**) over the last seven weeks and in total over twelve weeks were measured in DIAMOND mice with (white) or without (dark) 2-HOBA (1 g/L). (**C**) Body weights over time, (**D**) body weights at study end, and (**E**) liver to body weight ratios with (▪) or without (●) 2-HOBA were also measured. (**F**) Representative gross morphology of livers from control and 2-HOBA-treated DIAMOND mice. Scale bar = 3 mm. * *p* ≤ 0.05, ** *p* ≤ 0.01 by unpaired Student *t*-test.

**Figure 4:**
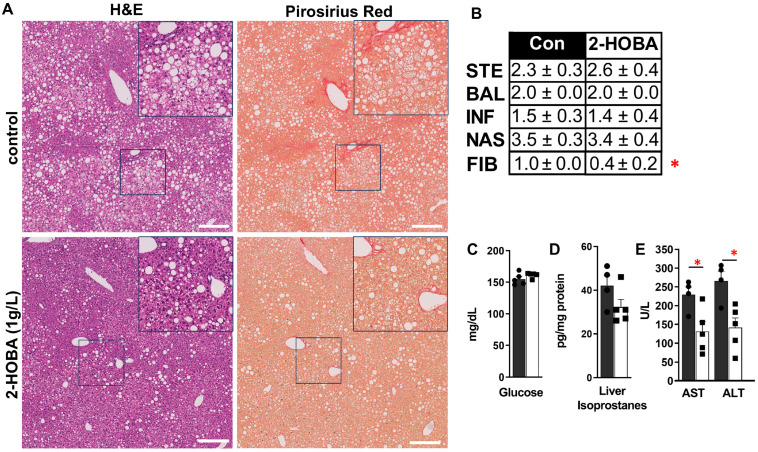
Effects of 2-HOBA on MASH severity in DIAMOND mice. (**A**) Left are H&E- and right are picrosirius-red-stained images of control (top) and 2-HOBA-treated mouse livers. (**B**) Average component (steatosis, inflammation, ballooning, and fibrosis) and composite histology scores for control and 2-HOBA-treated mice. (**C**) Glucose, (**D**) liver isoprostanes, and (**E**) transaminase levels in DIAMOND mice with (black bars) or without 2-HOBA. Scale bar = 200 μm. * *p* ≤ 0.05 by unpaired Student *t*-test.

DIAMOND mice showed slightly reduced food and water intake in the 2-HOBA group (Figure 3A, B) but no significant differences in body weight or liver-to-body weight ratio (Figure 3C–E). Gross liver morphology between groups was similar (Figure 3F), but despite equivocal steatosis severity, mice treated with 2-HOBA exhibited significantly lower fibrosis scores (* *p* = 0.012) (Figure 4A,B) and were associated with a 24% decrease in liver IsoPs (r = 0.69, *p* = 0.04). 2-HOBA and untreated mice exhibited similar blood glucose levels (~150 mg/dL) (Figure 4C) and demonstrated trends (*p* = 0.132) towards lower liver IsoPs in the 2-HOBA group (Figure 4D), although the differences did not reach statistical significance. Serum AST and ALT levels in 2-HOBA-treated mice (Figure 4E) were significantly reduced versus untreated controls (AST * *p* = 0.025, ALT * *p* = 0.012).

### 3.3. Effects of 2-HOBA in STAM Mice

**Figure 5:**
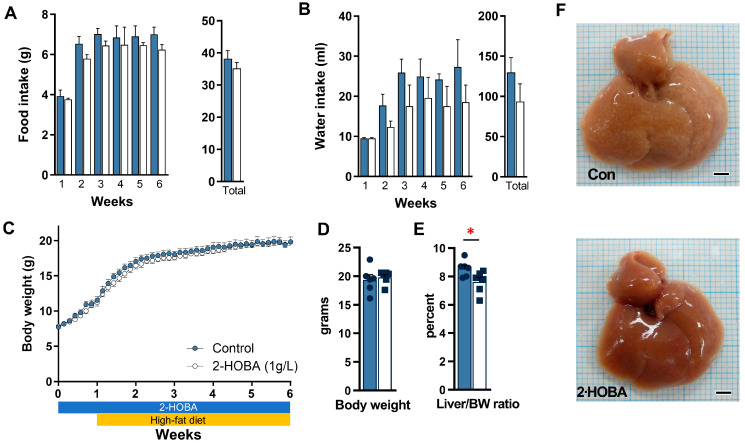
Food intake and water intake in STAM mice treated with 2-HOBA. (**A**) Food intake and water intake (**B**) over time and in total over six weeks were measured in STAM mice with (white) or without 2-HOBA (1 g/L). (**C**) Body weights over time, (**D**) body weights at study end, and (**E**) liver to body weight ratios with or without 2-HOBA were also measured. (**F**) Representative gross morphology of livers from control and 2-HOBA-treated STAM mice. Scale bar = 3 mm. * *p* ≤ 0.05 by unpaired Student *t*-test.

**Figure 6:**
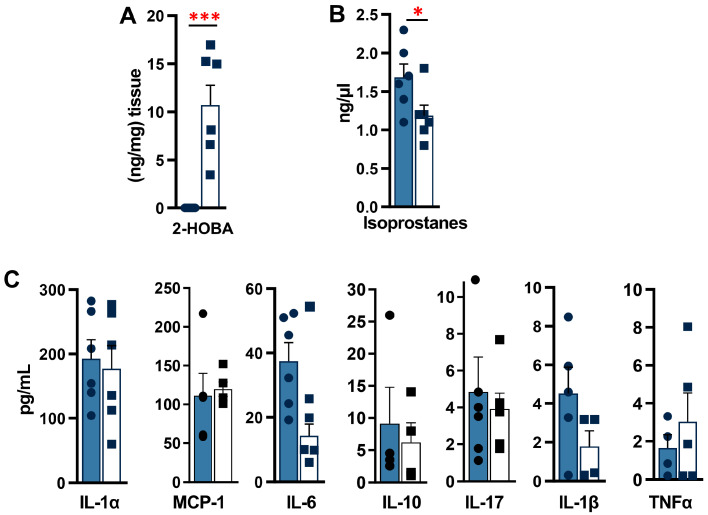
Effects of 2-HOBA on serum clinical chemistry and systemic inflammation in STAM mice. Fasting serum (**A**) 2-HOBA, (**B**) isoprostanes, and (**C**) the inflammatory cytokines IL-1α, MCP-1, IL-6, IL-10, IL-17, IL-1β, and TNFα measured by multiplexed ELISA. * *p* ≤ 0.05 and *** *p* ≤ 0.001 by unpaired Student *t*-test.

**Figure 7:**
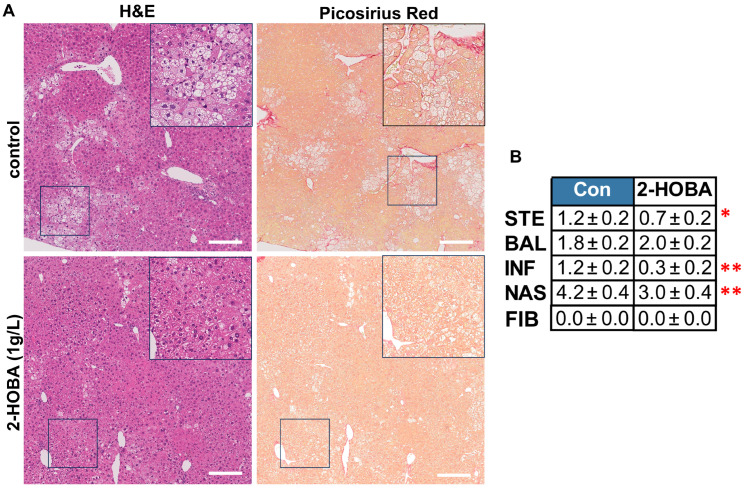
Effects of 2-HOBA on MASH severity in STAM mice. (**A**) Left are H&E- and right are picrosirius-red-stained images of control (top) and 2-HOBA-treated mouse livers. (**B**) Average individual component and composite scores for control and 2-HOBA-treated mice. Mag bar = 200 μm. * *p* ≤ 0.05, ** *p* ≤ 0.01 by unpaired Student *t*-test.

**Figure 8:**
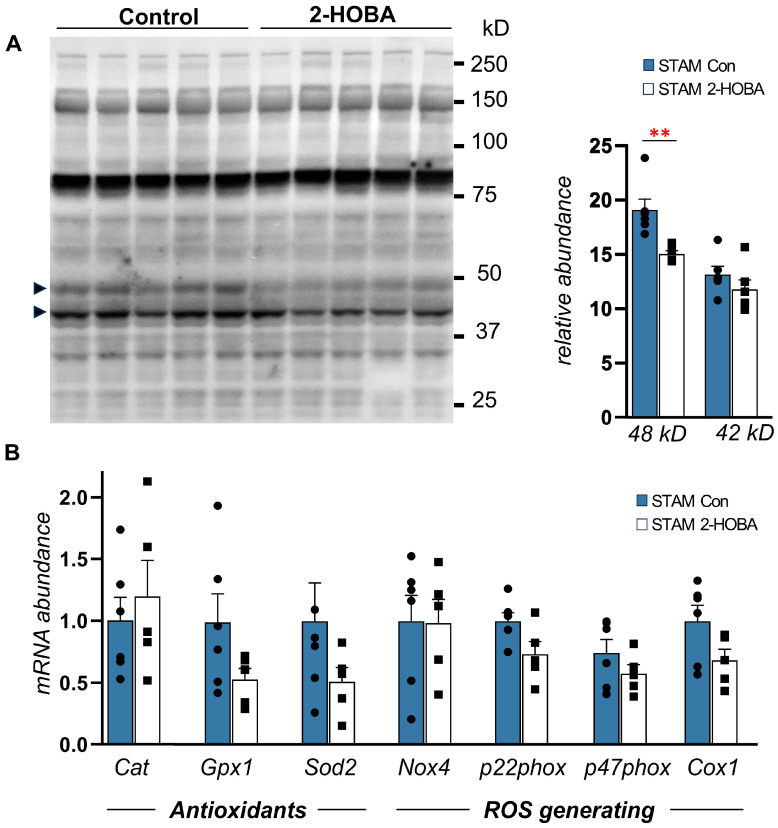
2-HOBA reduces dicarbonyl adducts in STAM mouse liver without altering the expression of key enzymes regulating ROS. (**A**) Immunoblot of liver total protein extracts from STAM control (blot left) or 2-HOBA-treated mice (blot right) probed with anti-D11 antibody. Bands of 42 kD and 48 kD were significantly differentially detected and are noted by arrows. (**B**) Relative levels of genes encoding antioxidant enzymes or reactive oxygen species (ROS)-generating proteins expressed in control (blue) and 2-HOBA-treated mouse livers. *Cat*, catalase; *Gpx1*, glutathione peroxidase 1; *Sod2*, superoxide dismutase 2; *Nox4*, NADPH oxidase 4; *P22phox*, protein 22 kd of phagocyte NADPH oxidase; *P47phox*, protein 47 kd of phagocyte NADPH oxidase; *Cox1*, cytochrome oxidase 1. *p* ≤ 0.05, ** *p* ≤ 0.01 by unpaired Student *t*-test.

The STAM Mouse Model Study showed significantly reduced liver mass and liver-to-body weight ratio in the 2-HOBA group by 11% (*p* = 0.04) (Figure 5D–F), with no significant differences in serum glucose and insulin levels (Supplemental Figure S1A,B). Liver 2-HOBA levels averaged 10 ng/mg tissue (Figure 6A), and these were associated with a 30% reduction in serum IsoPs (*p* = 0.05, oxidative stress marker) (Figure 6B) and improved AST levels, with no change in serum ALT (Supplemental Figure S1C) or serum TG levels (Supplemental Figure S1D). There were no significant differences in cytokine levels, but some trends were observed (Figure 6C), such as decreased IL-6 (*p* = 0.11) and IL-1β (*p* = 0.15). Histopathology demonstrated reduced steatosis and better-defined hepatic lobules in the 2-HOBA group (Figure 7A), with a significantly lower NAS in the 2-HOBA group (3.0 vs. 4.2 in controls, *p* = 0.03), lower inflammation (0.3 vs. 1.1, *p* = 0.01), and lower steatosis (0.7 vs. 1.2, *p* = 0.05). Molecular analysis showed decreased IsoLG-lysyl adducts in the 2-HOBA group (Figure 8A). The gene expression changes shown in Figure 8B demonstrated no changes in mRNAs encoding catalase (*Cat*), with trends towards lower glutathione peroxidase 1 (*Gpx1*; *p* = 0.13) and phagocyte NADPH-oxidase subunit 22 (p22phox; *p* = 0.07), as well as reduced expression of superoxide dismutase 2 (*Sod2*; *p* = 0.25), phagocyte NADPH-oxidase subunit 47 (*p47phox*; *p* = 0.329), and cytochrome c oxidase subunit 1(*Cox1*; *p* = 0.13). To investigate the effects of 2-HOBA on AKT/GSK3β signaling in STAM mice, we probed levels of total and phosphorylated GSK3β and AKT by immunoblot (Supplemental Figure S2). Mouse livers after 2-HOBA treatment showed significantly increased ratios of pSer473 AKT/total AKT (* *p* = 0.015) and pSer9 GSK3β/total GSK3β (*** *p* = 0.001).

In summary, 2-HOBA treatment showed promising effects in both DIAMOND and STAM mouse models of MASLD manifested by reduced liver mass and improved liver function markers, decreased oxidative stress and inflammatory markers, improved histopathological features of MASH, and enhanced AKT/GSK3β signaling, like metformin’s effects [40]. These findings suggest that 2-HOBA may have therapeutic potential in MASLD progression, consistent with previous studies on dicarbonyl scavengers in related metabolic disorders.

## 4. Discussion

This study investigated the abundance of isolevuglandin (IsoLG) adducts in humans with Metabolic-Associated Steatohepatitis (MASH) and evaluated the efficacy of 2-hydroxybenzylamine (2-HOBA) in mitigating MASH development in two preclinical diabetic MASH-HCC models: the DIAMOND model, an isogenic B6/I129 hybrid strain fed a Western diet with high-fructose–sucrose solution, exhibiting hypertriglyceridemia, and the STAM, chosen for its comprehensive representation of metabolic dysfunction, including obesity, insulin resistance, hyperglycemia, dyslipidemia, adipose tissue dysfunction, and adipokine imbalance. This study demonstrated the efficacy of 2-HOBA treatment manifested by significant reductions in liver weight to body weight ratio (Figure 5E), decreased NAFLD activity score (NAS) of hepatic steatosis (Figure 7B), attenuated NAS inflammation (Figure 3E), and a significantly decreased fibrosis score (Figure 4A,B), but with no significant reduction in serum cytokines (Figure 6C) or genes encoding liver antioxidants/ROS-generating enzymes (Figure 8B). Interestingly, these improvements were accompanied by increased liver AKT Ser473 and GSK3β Ser9 phosphorylation in 2-HOBA-treated mice, with potential effects on hepatic glucose metabolism and improved glucose homeostasis and insulin sensitivity.

The accumulation of TG in the liver stems from an increased influx of free fatty acids (FFAs) primarily from adipose tissue lipolysis (60%) and to a lesser degree from de novo lipogenesis (25%) and uptake of chylomicron dietary FFAs (15%) [41]. TG can be removed from liver hepatocytes through FFA oxidation or upon export as very-low-density lipoprotein (VLDL). In a state of lipid oversupply, ROS and other reactive intermediates are generated that react with lipids, proteins, and other biomolecules, leading to DE formation. The DE attacks rate-limiting enzymes in fatty acid (FA) metabolism such as carnitine palmitoyl transferase 1 (CPT1), monounsaturated FA formation (stearoyl-CoA desaturase; SCD), TG synthesis (diglyceride acyltransferase; DGAT), fatty acid synthesis (fatty acid synthase; FASN, and acetyl-CoA carboxylase; ACC), and lipoprotein assembly (microsomal triglyceride transfer protein; MTTP), potentially causing injury to hepatocytes. Similar injury could also occur upon DE modification of transcription factors (TFs) such as sterol regulatory element-binding protein-1c (SREBP-1C) and peroxisome proliferator-activated receptor-α a (PPARα) and -γ (PPARγ). Additionally, the metabolic dysregulation associated with hepatic steatosis, including insulin resistance and oxidative stress, can further exacerbate the production of DEs through various mechanisms, such as increased glycolysis, impaired detoxification pathways, and disruption of redox homeostasis. Collectively, these processes may cause injury to hepatocytes and perpetuate the formation of Des, creating an environment conducive to immune cell recruitment, stellate cell activation, and fibrosis. This hypothesis is supported by our findings of increased DE adducts in human MASH tissues (Figure 2), reduced serum IsoPs in 2-HOBA-treated STAM mice (Figure 6B), and decreased 48 kD immunoreactive band in 2-HOBA-treated STAM mice livers.

AKT (also known as Protein Kinase B) plays a central role in regulating glucose uptake, glycogen synthesis, and lipid metabolism, and cell survival with phosphorylation at Ser473 is a key marker of insulin signaling and its activation. Glycogen synthase kinase 3 (GSK3) is a multifunctional serine/threonine kinase with two isoforms, GSK3α and GSK3β, that regulate glycogen synthesis and glucose metabolism, with GSK3β being particularly implicated in liver diseases. GSK3β is a major kinase in MASH and a potential driver of lipotoxic inflammation that is pathogenically activated in numerous inflammatory conditions across multiple organs. Regulation of GSK3B activity occurs primarily through phosphorylation at serine 9 (pSer9 GSK3β), with increased pSer9 GSK3β being inhibitory [42]. Phosphorylation of serine 9 on GSK3β is directly regulated by the phosphorylation of serine 473 on Akt, meaning that when Akt is activated by phosphorylation at Ser473, it then phosphorylates GSK3β at Ser9, effectively inhibiting GSK3β activity [43,44]. Essentially, Ser473 Akt phosphorylation acts as an upstream signal that leads to Ser9 GSK3β phosphorylation and inactivation of GSK3β.

The liver is exposed to a number toxic compounds that must be detoxified but leads to the production of ROS. Toxicants such as carbon tetrachloride and ethanol are associated with oxidative injury to the liver [45]. The formation of IsoLG protein adducts in the liver was observed in rats given an oral gavage of a lipid and carbon tetrachloride. Four hours after the oral gavage, high levels of IsoLG protein adducts were observed in the liver [46], which can affect structural and functional modification proteins. Ethanol is a more common toxicant for humans, and consumption increases the risk of alcoholic fatty liver disease. Alcohol consumption in mice for 39 days led to an elevation in IsoLG protein adduct levels in a dose-dependent manner [47] and contributed to ethanol-mediated liver injury via a TNFR1/CYP2E1-dependent, but cyclooxygenase-independent, mechanism in the mouse liver. We have shown in C57BL/6J mice using the NIAAA model that pre-treatment with 2-HOBA for 14 days at 1.0 g/L prior to ethanol injury reduced liver injury, ameliorated the significant increases in hepatic IsoPs and IsoFs, and additionally decreased kidney F_2_-isoprostane formation (unpublished data). Further, we have shown that ethanol injury activated a robust hepatic overexpression of genes regulating oxidative stress, redox status, iron handling, xenobiotic metabolism, and lipopolysaccharide (LPS)-mediated RXR activation, whereas 2-HOBA ameliorated the expression of these pathways in the liver (unpublished data).

Treatment of cultured hepatic stellate cells (cells that drive hepatic fibrosis) with low doses of iso-LG induces cell activation and transformation to a profibrotic phenotype [16] and the expression of α-smooth muscle actin and cytokines, which are phenotypes of fibrosis and inflammation involved in MASLD. Likewise, increased electrophile stress and oxidized lipid peroxidation protein products (oxLPPs) are prominent in hepatocytes loaded with fatty acids (FAs) [48]. While in cultured cells these iso-LGs sustained activation of ERK and JNK signaling pathways, our in vivo data from 2-HOBA-treated STAM mice suggest that enhanced AKT/GSK3β signaling is a molecular feature of IsoLG scavenging. Further experiments are needed to establish which protein, DNA, or other biomolecular targets are responsible for this effect.

Recent findings on aspirin’s effects in MASLD patients provide context for potential 2-HOBA efficacy. A recent Phase 2 trial in 80 adults with MASLD, without cirrhosis, showed that one daily dose of aspirin, acetylsalicylic acid (81 mg), for 6 months reduced liver fat content by 6.6% versus placebo [49]. In preclinical studies, aspirin has been shown to exhibit anti-inflammatory effects in the liver through inhibition of cyclooxygenase enzymes and platelet-derived growth factor signaling. Aspirin also reduces bioactive lipid formation and DE availability via release of FAs from phospholipids and TGs [50], inhibits long chain 3-hydroxyacyl-coA dehydrogenase-mediated FA β-oxidation [51], and activates AMP-activated protein kinase A [52]. Aspirin has also been shown to reduce VLDL production [53] and inhibit elevated lipoprotein lipase (Lp(a)) [54] thereby altering hepatic availability of FFAs and potentiating accumulation of oxLPPs. Aspirin has been shown to inhibit the phosphorylation of Akt at Ser473 and decrease the activation of Akt signaling [55]. This effect is often observed in cancer cells, where aspirin can potentially suppress tumor growth by modulating this pathway [56]. Given the structural similarities between aspirin and 2-HOBA, and their favorable safety profiles [22,23,24,57], further studies on 2-HOBA in human MASLD may be warranted.

This study has limitations. We were only able to study male mice. While human MASLD is more prevalent in men than in women [58], it is unclear whether there are sex-associated differences in MASLD pathology or response to treatment. Future studies should investigate the efficacy of 2-HOBA in both sexes. There also appeared to be potential associated differences in MASH pathology and 2-HOBA responses that were not further investigated. Several potential factors contributing to these discrepancies, such as genetic background, diet, age, and extent of MASLD severity differ between the STAM and DIAMOND models. Future studies attempting to standardize these variables as much as possible to allow for more comparable results may be warranted. Additional approaches utilizing primary hepatocytes in in vitro studies, co-culture experiments with hepatocytes and other cell types, and/or additional biomarker analyses may be warranted. 2-HOBA administration began prior to MASH development, demonstrating a protective effect rather than a therapeutic one. An alternative approach would be to treat mice with MASH in a therapeutic setting, where MASLD and/or MASH have already developed. In these studies, careful consideration is warranted regarding dosing, timing, and duration of 2-HOBA exposure as MASH can develop into cirrhosis and hepatocellular carcinoma in both STAM and DIAMOND models. Furthermore, preclinical studies may not fully recapitulate the complex pathophysiology of human MASLD and MASH. Factors such as inter-individual variability, drug–drug interactions, and unforeseen adverse events can significantly impact the clinical efficacy and safety of therapeutic interventions. First-in-human studies already have established 2-HOBA as safe and well-tolerated, with defined pharmacokinetics [59,60]. Clinical studies with 2-HOBA are presently being evaluated for efficacy in preventing Alzheimer’s disease, rheumatoid arthritis, familial hypercholesterolemia, and pulmonary arterial hypertension. Finally, the study does not address the potential for 2-HOBA to reverse established MASH pathogenesis. Some drug classes, such as FGF21 analogs, have shown potential to reverse fibrosis, with reduction in liver fibrosis biomarkers alongside improvements in liver fat and inflammation [61]. These drugs, like 2-HOBA, may offer broader metabolic benefits by multiple mechanisms including improving insulin sensitivity and reducing dyslipidemia, potentially contributing to further improvements in liver health.

## 5. Conclusions

In conclusion, our data support the potential of 2-HOBA, an electrophile scavenger, as a strategy to reduce MASLD inflammation and steatosis associated with progression to MASH. Future studies should investigate 2-HOBA efficacy in both sexes, evaluate 2-HOBA treatment after the onset of clinical MASH to determine its therapeutic value, and further explore the molecular mechanisms underlying 2-HOBA’s effects, particularly its impact on AKT/GSK3β signaling in liver tissue. These findings pave the way for potential clinical trials to assess 2-HOBA’s efficacy in human MASLD patients.

## Data Availability

The datasets generated and/or analyzed during the current study are available from the corresponding authors on reasonable request.

## Supplementary Materials

The following supporting information can be downloaded at: www.mdpi.com/article/10.3390/nu17040610/s1, Figure S1: Effects of 2-HOBA on the liver; Figure S2: 2-HOBA enhances insulin signaling in STAM mouse liver.

## Abbreviations

2-HOBA, 2-hydroxybenzylamine, 4-HNE 4-oxo-2-nonenal; ACC, acetyl-CoA carboxylase; AKT, viral v-akt gene product/serine/threonine protein kinase; AMP, adenine mononucleotide phosphate; CAT, catalase; COX1, cyclooxygenase 1; CPT1, carnitine palmitoyl transferase 1; dC, deoxycytidine; DE, dicarbonyl electrophile; DGAT, diglyceride acyltransferase; DIAMOND, Diet-Induced Animal Model of Nonalcoholic Fatty Liver Disease; ERK, Extra-cellular Signal Regulated Kinase; FA, fatty acid; FASN, fatty acid synthase; GPX1, glutathione peroxidase; GSK3β, glucose synthase kinase β; HDL, high-density lipoprotein; IsoLG, isolevuglandin; IsoPs, isoprostanes; JNK, jun kinase; Ldlr, low-density lipoprotein receptor; MASLD, metabolic-associated steatotic liver disease; MASH, Metabolic-Associated Steatohepatitis; MDA, malondialdehyde; MTTP, microsomal triglyceride transfer protein; MDA, malondialdehyde; NOX4, nitric oxide synthase 4; HCC, hepatocellular carcinoma; OxLPPs, oxidized lipid peroxidation protein products; p47phox, protein 22 kd of phagocyte NADPH oxidase; p22phox, protein 47 kd of phagocyte NADPH oxidase; PCR, polymerase chain reaction; PE, phosphatidylethanolamine; PGH2, prostaglandin endoperoxide; PPARα, peroxisome proliferator-activated receptor-α; PPARγ, peroxisome proliferator-activated receptor-γ; ROS, reactive oxygen species; SCD, stearoyl-CoA desaturase; SOD2, superoxide dismutase 2; SREBP-1C, sterol regulatory element-binding protein-1c; STAM, Stelic Animal Model.
