# Supplementary material for: Reactive Dicarbonyl Scavenging with 2-Hydroxybenzylamine Improves MASH
Source: Nutrients. 2025 Feb 7;17(4):610. doi: 10.3390/nu17040610 (PMC11858443; doi:10.3390/nu17040610)
Supplement: Supplementary file 1 [file nutrients-17-00610-s001.zip › Supplemental FIG Legends.pdf]

Supplemental FIG. S1. Effects of 2-HOBA on the liver. Fasting levels of (A) blood glucose, (B)serum insulin, (C) liver transaminases AST and ALT, and (D) serum triglycerides from STAM mice without (Control) and with 2-HOBA (1 g/L).

Supplemental FIG. S2. 2-HOBA enhances insulin signaling in STAM mouse liver. (A) Immunoblots of liver total protein from STAM mice without (Control) and with 2-HOBA (1 g/L). (B) The AKT pSer473/ total AKT ratio and (C) GSK3β pSer9/ total GSK3β ratio are increased with 2-HOBA. *$p < 0.01$, **$p < 0.01$ by paired Student t-test.
