# Supplementary figures and images for: Reactive Dicarbonyl Scavenging with 2-Hydroxybenzylamine Improves MASH
Source: Nutrients. 2025 Feb 7;17(4):610. doi: 10.3390/nu17040610 (PMC11858443; doi:10.3390/nu17040610)

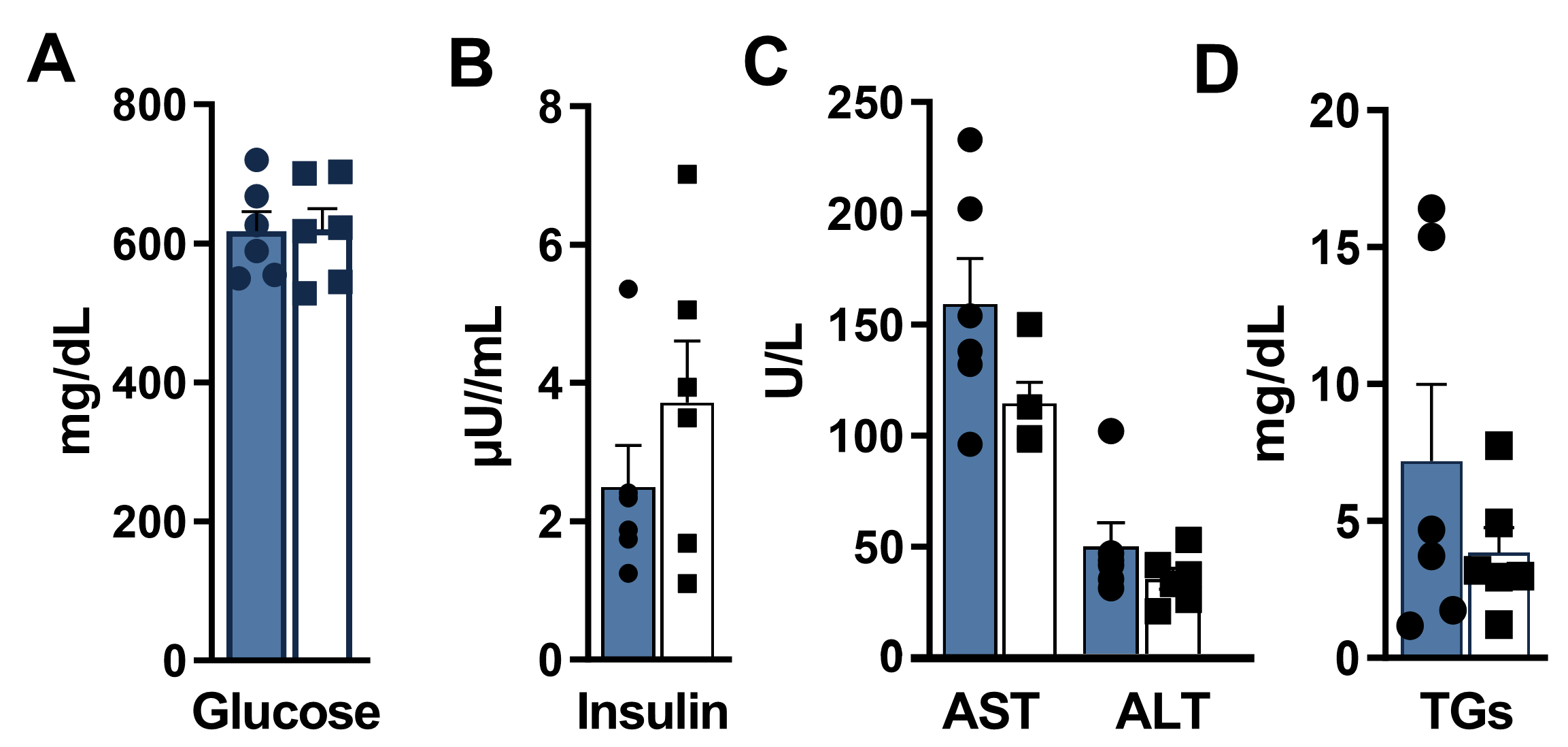

Supplement: Supplementary file 1 [file nutrients-17-00610-s001.zip › Supplemental Figure S1.tif]

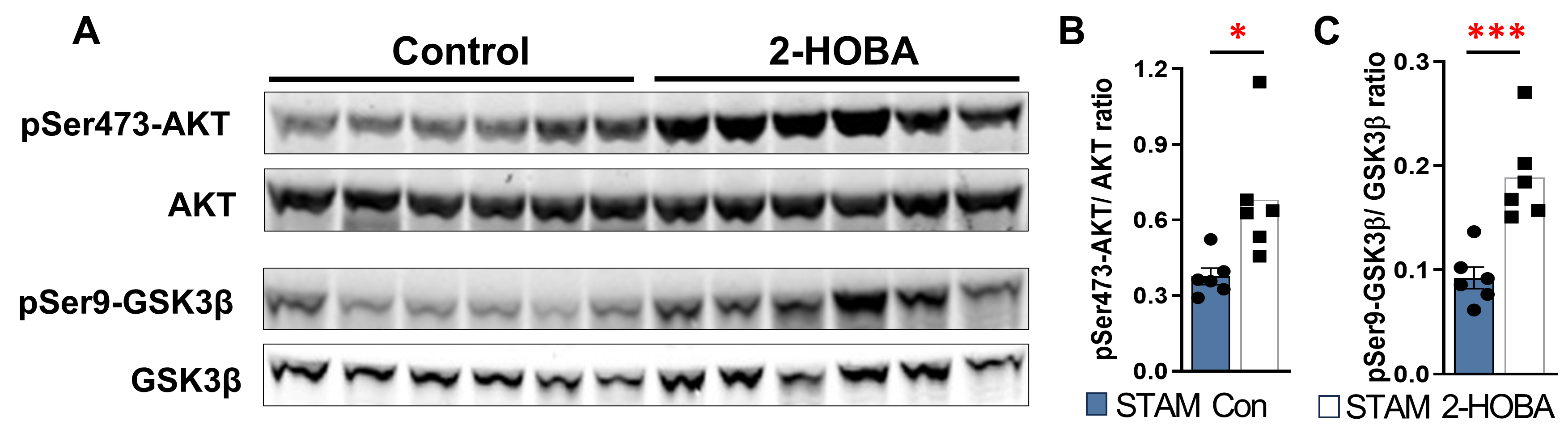

Supplement: Supplementary file 1 [file nutrients-17-00610-s001.zip › Supplemental Figure S2.tif]
